# A systematic review of neuroimaging epigenetic research: calling for an increased focus on development

**DOI:** 10.1038/s41380-023-02067-2

**Published:** 2023-04-25

**Authors:** Esther Walton, Vilte Baltramonaityte, Vince Calhoun, Bastiaan T. Heijmans, Paul M. Thompson, Charlotte A.M. Cecil

**Affiliations:** 1 Department of Psychology, University of Bath, Bath, UK; 2 Tri-institutional Center for Translational Research in Neuroimaging and Data Science, Georgia State University, Georgia Institute of Technology, Emory University, Atlanta, GA, USA; 3 Molecular Epidemiology, Dept. of Biomedical Data Sciences, Leiden University Medical Center, Leiden, The Netherlands; 4 Imaging Genetics Center, Mark and Mary Stevens Neuroimaging & Informatics Institute, Keck School of Medicine of the University of Southern California, Marina del Rey, CA, USA; 5 Department of Child and Adolescent Psychiatry/Psychology, Erasmus University Medical Center Rotterdam, Rotterdam, The Netherlands; 6 Department of Epidemiology, Erasmus University Medical Center Rotterdam, Rotterdam, The Netherlands

## Abstract

Epigenetic mechanisms, such as DNA methylation (DNAm), have gained increasing attention as potential biomarkers and mechanisms underlying risk for neurodevelopmental, psychiatric and other brain-based disorders. Yet, surprisingly little is known about the extent to which DNAm is linked to individual differences in the brain itself, and how these associations may unfold across development – a time of life when many of these disorders emerge. Here, we systematically review evidence from the nascent field of Neuroimaging Epigenetics, combining structural or functional neuroimaging measures with DNAm, and the extent to which the developmental period (birth to adolescence) is represented in these studies. We identified 111 articles published between 2011–2021, out of which only a minority (21%) included samples under 18 years of age. Most studies were cross-sectional (85%), employed a candidate-gene approach (67%), and examined DNAm-brain associations in the context of health and behavioral outcomes (75%). Nearly half incorporated genetic data, and a fourth investigated environmental influences. Overall, studies support a link between peripheral DNAm and brain imaging measures, but there is little consistency in specific findings and it remains unclear whether DNAm markers present a cause, correlate or consequence of brain alterations. Overall, there is large heterogeneity in sample characteristics, peripheral tissue and brain outcome examined as well as the methods used. Sample sizes were generally low to moderate (median *n*_all_ = 98, *n*_developmental_ = 80), and attempts at replication or meta-analysis were rare. Based on the strengths and weaknesses of existing studies, we propose three recommendations on how advance the field of Neuroimaging Epigenetics. We advocate for: (1) a greater focus on developmentally oriented research (i.e. pre-birth to adolescence); (2) the analysis of large, prospective, pediatric cohorts with repeated measures of DNAm and imaging to assess directionality; and (3) collaborative, interdisciplinary science to identify robust signals, triangulate findings and enhance translational potential.

## Introduction

Large-scale consortium efforts as well as advances in technology and methods have made it increasingly feasible to map the relationship between brain measures and a wide range of mental health outcomes in unprecedented detail. These neural correlates are shaped by both genetic and environmental influences: twin-based heritability estimates range between 60–80% for brain volume with slightly lower values for measures of white matter integrity (14–64%), resting-state functional connectivity (15–42%) and task-based functional MRI (40–65%) [1, 2]. Although both genetic and environmental factors (e.g., stress, substance use, especially when experienced in early life) clearly influence the developing brain, we do not yet understand how these factors jointly shape brain structure and function, and downstream risk for neurodevelopmental, psychiatric and other brain-based disorders. As many of these disorders have early origins (e.g. with roughly one third of people developing mental illnesses before the age of 14 and one half before the age of 18 [3]), it is particularly important to understand these processes from a developmental perspective.

Epigenetic mechanisms that regulate gene expression, such as DNA methylation (DNAm, Box 1), have gained increasing attention in the neuroimaging field as a potential mechanism mediating—or a biomarker of—genetic and environmental influences on the developing brain. DNAm is a key process involved in development through its role for example in embryogenesis [4, 5], cell specification [6], genomic imprinting and X-chromosome inactivation [7, 8], and ageing [9].

Growing excitement regarding DNAm within mental health research stems from evidence that: (i) DNAm is influenced by genetic and environmental factors (e.g. psychosocial exposures, diet), as early as pregnancy [10]; (ii) DNAm plays an essential role in normative development, including brain maturation and function [11]; and importantly that (iii) disruptions in DNAm patterns associate with numerous brain-based disorders [12], including neurodevelopmental (e.g. autism spectrum disorder), psychiatric (e.g. depression, schizophrenia) and neurodegenerative (e.g. Alzheimer’s disease) disorders.

Notably, DNAm shows huge promise as a biological marker for disease prediction, early detection and risk stratification, especially for use in peripheral tissues that are more readily accessible in humans even if not causal for the phenotype of interest. For example, it is already possible to estimate a range of exposures, traits and health outcomes based on peripheral DNAm patterns alone (e.g., age, smoking, BMI) [13], and to detect certain diseases sooner and more accurately when complementing conventional diagnostic methods [14–16], including brain-based disorders [17], leading to improved clinical care [18, 19].

A key outstanding question is to what extent peripheral DNAm can inform about individual differences in the living brain—the most relevant organ for the study of neurodevelopmental, psychiatric and other brain-based disorders— and how associations may vary by developmental stage. The nascent field of *neuroimaging epigenetics* (Box 1) aims to fill these gaps by uniting scientists from different disciplines, such as psychiatry, neuroimaging and developmental biology to characterize epigenetic correlates of the brain in vivo. So far, a rapidly growing number of studies have reported associations between DNAm patterns and various aspects of brain structure and function. However, findings across studies have been largely inconsistent [20]. A number of reasons have been proposed for this, including heterogeneity with regard to study design (prospective versus cross-sectional), sample characteristics (e.g., population-based vs clinical), analytic approaches used (e.g., candidate versus genome-wide) and health outcomes examined.

Crucially and less commonly acknowledged, different studies have investigated widely different ages and developmental stages, spanning birth to old age. Both epigenetic patterns and the brain are highly dynamic from birth to adulthood [21, 22], and both change at different, often non-linear rates across development (i.e., following a log-linear pattern with faster rates in early development [23, 24]). This variation might result in time-specific associations. For example, DNAm patterns at birth, but not in childhood, have been found to be a stronger predictor of ADHD symptoms in childhood [25]. In another study, glucocorticoid exposure was found to exert a stronger and more lasting effect on DNAm patterns in hippocampal neuronal progenitor cells, if this exposure occurred earlier in neurogenesis (i.e., proliferation stage as opposed to post-differentiation [26]). Evidence from both studies illustrate how DNAm at specific developmental periods may be more sensitive to exposures or more predictive of future brain-based outcomes. Hence, the choice of when to assess DNAm may influence whether associations are identified or not. Several mechanisms could explain these time-specific findings. For instance, DNAm variation increases in variability [27–29] and decreases in heritability [30, 31] as people age, pointing towards stronger environmental (or stochastic) influences later in life. In addition, timing effects may in part reflect tissue and cell-type differences between commonly examined biospecimens at different ages (e.g., cord blood at birth containing multipotent cells that are scarcely found in peripheral blood later in life), which may also influence the degree of peripheral-brain concordance over time. Finally, important confounders may play a larger role at specific developmental time points (e.g., stronger gender effects in adolescence). Any of these factors (e.g., rate of change, genetic and environmental influences, tissue, confounders) may impact variations across development and contribute to heterogeneity between studies. However, currently such a perspective is largely lacking in the field of neuroimaging epigenetics.

To address this gap, we systematically reviewed emerging research across the lifespan, combining structural or functional neuroimaging measures with DNAm, and evaluated to what extent the developmental period (birth to adolescence) is represented in these studies. Our aim was three-fold: (i) to provide an overview of the current state-of-the-art in this new research field; (ii) to discuss current challenges; and (iii) to provide concrete recommendations on how to address these in the future. We argue that it is important to set standards now while the field of neuroimaging epigenetics is still in its infancy.

## Reviewing the Current State of Neuroimaging Epigenetics From a Development Perspective

### Developmental periods are underrepresented

**Fig. 1:**
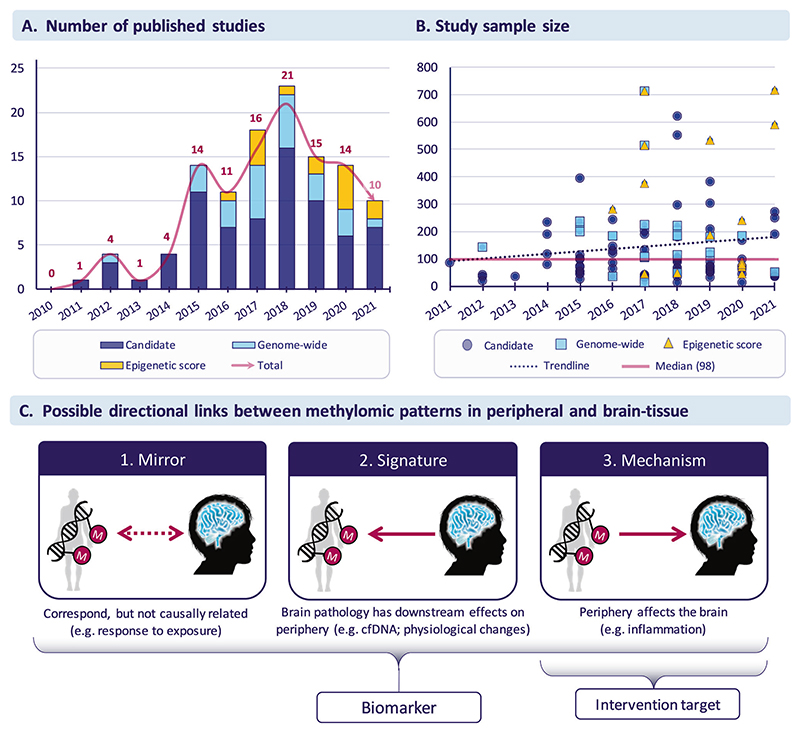
Research studies in the field of neuroimaging epigenetics. **A** Number of published studies between 2011 and 2021. **B** Sample sizes across studies. **C** Possible directional links between methylomic patterns in peripheral and brain tissue. Of note, not shown but equally possible is the absence of a relationship between peripheral and brain-tissue methylation (i.e., with both, neither or only one of these DNAm measures influencing neuroimaging traits). In addition and like DNAm in peripheral tissues, brain tissue methylation is highly heterogenous both spatially (i.e., between brain regions and cell types) and temporally. Hence, any one of the proposed relationships might be true for some but not other brain regions or life stages.

Overall, we identified 111 articles, published between 2011–2021 (Fig. 1A;for methods, see supplemental materials). Out of the total number of studies, only a minority (21%) focused on developmental periods, such as neonatal (*n* = 6), childhood (*n* = 6) and adolescence (*n* = 16; Table 1). Overall, most of the studies were based on adult samples (*n* = 85) and very few on old age (*n* = 8 in vivo and 2 postmortem studies).

Most studies (75%) investigated DNAm-brain associations in the context of health and behavioral outcomes, such as depression and anxiety (*n* = 25), psychotic disorders (*n* = 20), post-traumatic stress disorder (*n* = 8), or neurodegenerative and ageing-related traits (*n* = 7; Table 1, right panel), but several publications were based on the same datasets. In contrast, only 25% of studies considered how environmental influences such as childhood trauma or other forms of stress might affect DNAm-brain associations. For developmental studies in particular, the pattern was somewhat different: fewer studies assessed health or behavioral outcomes (57%) and a greater number (57%) examined environmental effects. Almost half of all studies (*n* = 48) included genetic data in their analysis, suggesting a large interest in the unique versus joint effects of genetic and epigenetic variation on brain-based phenotypes. For details, see SM Tables 1 and 2.

Although both DNAm and neuroimaging are high-dimensional data types, sample sizes across studies were on average modest, with a median sample size of 98 (range 14–715; Fig. 1B). Sample size tended to increase continuously with the year of publication (*ρ* = 0.14). For developmental studies, the median sample size was 80 (range 33–715), which was slightly smaller than for studies overall. The largest of the identified studies found no association between brain aging and epigenetic aging [32].

Overall, studies focused primarily on clinical samples (45%), followed by population-based cohorts (26%), high-risk groups (18%), convenience or community samples such as college students (10%), and twin and family samples (4%). However, most developmental studies were based on high-risk (39%) and population-based cohorts (35%) rather than clinical samples (17%). This could indicate that either (1) childhood and adolescence are developmental periods underrepresented in clinical research or that (2) clinical phenotypes examined in adults (e.g., schizophrenia, bipolar disorder) have not yet fully manifested at a younger age.

### A prospective study design, including repeated measures of DNAm or MRI is a key strength of developmental studies

Overall, most studies—although sometimes longitudinal in their overall design—featured a cross-sectional set-up, with DNAm and neuroimaging measures obtained at the same time point (85%). Of those that measured these variables at different time points, most obtained DNAm first and then data on brain structure or function. Diverging from the overall pattern, developmental studies were more likely to follow a prospective (57%) than cross-sectional design (43%). Follow-up periods ranged from weeks (most often in studies with neonatal samples [33, 34]) to decades [35].

Overall, eight studies (7%) measured either DNAm or MRI (or both) repeatedly, but none more than twice (e.g., 20–23, 36–38). Only one study investigated the effect of repeated measures of DNAm on MRI at the genome-wide level [35].

### The large degree of methodological and statistical heterogeneity impacts reproducibility

Overall, the majority of studies (67%) focused on candidate epigenetic markers, including candidate genes and selected DNAm sites (ranging from *n* = 1 to several hundred sites; sometimes followed up based on an initial epigenome-wide association study; EWAS). Seventy-one studies focused on *n* = 34 different genes, with *SLC6A4, OXTR* and *FKBP5* among the most prominent ones. However, even in studies that focused on the same gene, the exact genomic region investigated was often different.

Of those studies following a genome-wide approach (23%), most conducted a probe-level EWAS. However, in some of these studies, the number of DNAm sites were reduced by up to 90% before analysis, for example, based on a selection of highly variable probes [33, 39]. Only a minority of studies (11%) examined measures of epigenetic age acceleration (Box 1), which was investigated predominantly in studies of stress or age-related cognitive decline [40–45]. In developmental studies, the proportion of candidate and genome-wide studies was slightly more balanced (52% and 30%, respectively).

Only 11 studies (five of which were based on children and adolescents) included a replication step in an independent cohort—nearly all (*n* = 9) using a candidate gene or epigenetic score approach—and none performed a meta-analysis of multiple cohorts (SM Table 1). The most common covariates were age (*n* = 77) and sex (*n* = 72). Among the possible neuroimaging covariates, only a few studies reported accounting for site effects (*n* = 11), motion covariates (*n* = 13), or intracranial volume (*n* = 16). With regard to DNAm-relevant covariates, only ten studies reported controlling for array effects, with a slightly larger number correcting for batch effects (*n* = 16) and cell-type proportions (*n* = 21). Covariates were variable across studies and often selected based on statistical results (e.g., if they were significantly different across groups) rather than on a priori, theoretically informed approaches.

While overall, peripheral blood was by far the most studied tissue (*n* = 83;75%), followed by saliva (*n* = 23;21%), buccal (*n* = 5;5%) and postmortem brain tissue (i.e., in relation to postmortem MRI; *n* = 3;3%, including replication datasets), developmental studies investigated saliva (*n* = 11; 48%) more frequently than blood (n=9; 39%). This could be due to saliva sampling being a less invasive method, which may be chosen as a more appropriate option in younger participants. Very few studies measured DNAm in more than one tissue type. With regard to imaging methods, we observed a relative balance between studies using structural MRI (48%) and task-based fMRI (35%), followed by diffusion MRI (19%), resting-state fMRI (7%), and PET (5%). Akin to the predominant adoption of a candidate gene approach observed for DNAm, most MRI studies (67%) followed a region-of-interest (ROI) approach, primarily focusing on the hippocampus or the amygdala. This pattern did not seem to differ in developmental studies.

## Synthesizing Current Challenges

Developmental periods were underrepresented in neuroimaging epigenetics research, with only a fifth of studies focusing on these periods. Fewer developmental studies explored health and behavioral outcomes and a greater number examined environmental effects. We observed that developmental studies were more likely to be prospective, featuring repeated measures of DNAm or MRI, which is a key strength when assessing the role of DNAm and MRI in the development of psychopathology. We also observed a large degree of methodological and statistical heterogeneity across studies, which could impact reproducibility. Given the findings of the present review, in the following, we expand on three key challenges: the lack of developmentally oriented research, directionality of associations, and comparability across studies. We highlight concrete recommendations to move the field of neuroimaging epigenetics forward, building upon previous recommendations in the field of epigenetics [46–50].

### Lack of developmentally oriented research

Research involving children and adolescents is largely underrepresented in neuroimaging epigenetics research. While this may be less problematic for research interested in conditions linked to old age (e.g. neurodegenerative disorders), it is a key limitation in the context of psychiatric and behavioral disorders, which typically have neurodevelopmental origins and tend to emerge early in life [3]. In this respect, the early developmental period may be especially relevant for understanding how genetic and environmental factors influence brain structure and function, and, crucially, subsequent psychiatric risk. Furthermore, growing evidence suggests that DNAm changes dynamically over development, and that DNAm patterns contributing to psychiatric risk may also differ across time [51, 52]. Indeed, studies show that DNAm at birth tends to be more predictive of certain psychiatric outcomes than DNAm in childhood or late adolescence [25, 53, 54]. This implies that associations between DNAm and the brain, or the pattern of associations, may be time-dependent, and suggests that findings in adult samples may not be readily generalizable to other developmental stages.

### Establishing directionality of associations

The studies reviewed here broadly support an association between peripheral DNAm and brain structure or function. Yet, based on these findings, it is unclear whether DNAm markers present a cause or consequence of brain alterations or are due to other confounding variables. Disentangling the direction of effect is extremely difficult, as most imaging epigenetics research is based on cross-sectional, case-control samples. Different scenarios are possible. First, in line with a model proposed by Aberg et al. [55], peripheral epigenetic patterns might mirror those in brain tissue, possibly due to a common cause. Here, an exposure (such as smoking) might impact both peripheral DNAm as well as brain traits, resulting in a non-causal association between the two (“Mirror” model in Fig. 1C). Second, brain pathology may have downstream effects on peripheral processes affecting DNAm. For instance, changes in hypothalamic structure or function may impair metabolic or hormonal processes, which then leave a signature in peripheral DNAm (“Signature” model in Fig. 1C). In this case, the DNAm signature would not be causal for brain-based phenotypes or related psychiatric traits, but a downstream consequence of them. Last, the reverse could also be envisaged whereby peripheral processes, such as increased inflammation or vascular events, could alter brain traits via changes in DNAm (“Mechanism” model in Fig. 1C). In all these scenarios, DNAm markers could be used as a biomarker of disease (or risk thereof), but only in the “Mechanism” model could we use peripheral DNAm as an interventional target, so long as the brain is the causal tissue for the disease. These scenarios do not need to be mutually exclusive and animal studies so far provide independent evidence for all three scenarios [56–58]. Causal inference methods such as Mendelian Randomization (MR) provide an opportunity to assess causality in human data [59, 60], but to our knowledge only one study to date has used MR to investigate causal links between DNAm and MRI phenotypes [61].

### Comparability across studies

Studies were highly heterogeneous with respect to the design (e.g., longitudinal versus cross-sectional), sample characteristics (e.g., clinical, population-based), tissue (e.g., blood, saliva) and methodological and statistical approaches used (e.g., candidate gene versus genome-wide; diverging sets of confounders;variable preprocessing pipelines). Relatively few studies shared practices and common standards, which might be seen in other, more mature fields (e.g., neuroimaging, psychiatric genetics) [62–67]. Sample sizes were on the whole moderate (median *n*_all_ = 98, median *n*_developmental_ = 80) and only 10% of studies included a replication step in an independent cohort. This type of variability across studies poses a challenge when attempting to synthesize research findings.

Cross-tissue heterogeneity in particular is a major concern in epigenetic research related to brain disorders [49]. Studies of cross-tissue correlation based on biopsy or postmortem samples in general do not find large overall comparability between tissues, and only a small proportion of DNAm sites show cross-tissue correspondence [68, 69]. Equally important are concerns regarding cross-tissue heterogeneity across different *peripheral* tissues [50] that highlight the need to investigate which peripheral tissue might be most informative about the status of the brain. The choice of tissue might also address difficulties related to reduced compliance and heightened attrition when blood samples are required (compared to buccal swabs), especially in clinical populations or longitudinal samples.

## Ways Forward

### Recommendation 1: A greater focus on development

To truly understand associations between DNAm and the brain in the context of early environmental exposures and downstream outcomes such as psychiatric disorders, we call for a greater focus on developmental research. Studies are needed to map DNAm-brain associations at different, and ideally sensitive, developmental periods (e.g., birth, puberty). This could help to uncover time-specific links with exposures or outcomes, characterize directional changes in associations over time, and thereby have important implications for understanding, predicting and treating brain disorders that might only emerge later in development. For example, cord blood infusion is currently being trialed as a promising therapy for reversing brain damage in a number of child and adult disorders (e.g. cerebral palsy, brain injury, Parkinson’s disease [70, 71]). This is based on evidence that multipotent cord blood cells exert powerful neuroprotective and anti-inflammatory effects, raising the possibility that these cells may be more informative for the study, prediction and treatment of brain-based phenotypes than cells present at later developmental stages. Yet, how these multipotent cells relate to DNAm patterns at birth, and whether they could partially explain the inconsistent associations with brain structure and function across development, still needs to be investigated.

### Recommendation 2: The use of prospective, pediatric cohorts with repeated measures of methylation and imaging

To disentangle the directionality of effects, we need studies that collect repeated measures of DNAm and MRI data over time. In addition, relating DNAmto MRI patterns early in life could shed light on the role of epigenetic and brain variation in the onset and persistence of mental disorders. This ideally requires (i) prospective data in young individuals beginning *before* the onset of symptoms; (ii) the availability of repeated measures of both DNAm and brain imaging at different developmental periods; and (iii) longitudinal follow-ups into adulthood. There are few cohorts worldwide with a study design that can address these questions (see Table 2 for a non-exhaustive list of cohorts). Using this set-up within the ALSPAC cohort, we were able to show, for example, that DNAm at birth is more predictive of later ADHD symptoms (measured repeatedly between ages 7 and 15) than DNAm measured at age 7 [72]. In relation to the brain, we could also show that the majority of DNAm sites at birth and age 7, are not stably predictive of amygdala:- hippocampal volume, measured at age 18 [35].

To further strengthen causal inference or in cases where no such data is available, two-sample MR [73] can be applied, ideally using developmentally and tissue-specific genetic instruments (e.g., single nucleotide polymorphisms that are associated with early brain development). For example, Korologou-Linden [74] found that Alzheimer disease risk has an age-dependent effect on a range of cortical and subcortical brain measures that starts in mid-life but not earlier in development. Using data for methylation quantitative trait loci derived from prefrontal cortex tissue, Hatcher et al. [75] applied MR and found that DNAm may putatively mediate effects of genetic variants on traits, such as schizophrenia. Moreover, it is important to keep an open mind regarding the specific epigenetic mechanism involved. DNA methylation changes may well not be causal in themselves but mark other regulatory processes like changes in transcription factor binding or in histone modifications.

### Recommendation 3: Push towards collaborative science

To improve comparability across studies, we recommend, where possible, to harmonize DNAm and MRI preprocessing pipelines [76], incorporate meta-analytic approaches [77] or to replicate the original study findings, especially in the context of candidate gene studies [67]. We encourage the adoption and further development of already established standards in the field of neuroimaging, such as *Brain Imaging Data Structure* (BIDS [62]) or COBIDAS [63], as well as recommended best practices in genetics (or related ‘omics’ areas; e.g. [64–67]). This will help to increase sample size, maximize statistical power to detect small effects and weed out false positives. For instance, researchers interested in combining DNAm and neuroimaging may draw on the information provided in this review to identify potential replication samples (see SM Table 1). A recent successful example of collaborative science comes from the ENIGMA-Epigenetics consortium which combined 3,337 samples from 11 cohorts [77]. The authors examined epigenome-wide associations with three subcortical volumes and identified two CpGs linked to the hippocampus, each explaining 0.9% of the phenotypic variance. These results point to small effect sizes in the relationship between individual peripheral DNAm markers and subcortical volumes, which might indicate limited value of single CpGs as biomarkers for brain structure. However, small effects might still be of mechanistic relevance, providing evidence for causality (see recommendation 2).

In line with lessons learned in the field of psychiatric genetics, a candidate gene approach should be well justified, for example, as a follow-up of EWAS results or functional studies (e.g., genetic knock-out, in vitro results). While systematic reviews might also provide such evidence, we recommend triangulation of evidence derived from multiple and complementary approaches (e.g., assessing publicly deposited (epi-)genome-wide data or summary results in databases such as Gene Expression Omnibus or the EBI GWAS catalog; for a more comprehensive list, see [78]) to overcome limitations such as publication bias.

At the same time, we should further develop the use of epigenetic scores and global measures such as brain ageing. We also need to expand multivariate data-dimension reduction techniques such as parallel ICA to uncover DNAm-brain relationships that might remain hidden when applying univariate approaches [79, 80].

Furthermore and of particular relevance for candidate gene studies, an increased focus on interdisciplinary collaborations will be needed to characterize DNAm-brain associations more robustly and comprehensively, and to triangulate findings generated using different approaches. For example, collaborating with experimental researchers could facilitate the use of tools for targeted gene-or region-specific epigenetic editing in animal models, which would help to evaluate functional consequences of localized DNAm changes on brain development and downstream outcomes such as psychiatric disorders [81]. At the same time, employing in vitro models may help to characterize the role of gene- or region-specific DNAm markers associated with brain-based traits at a cellular level [82].

### The MIND consortium

To address these challenges, we established a platform for advancing the field of neuroimaging epigenetics—the Methylation, Imaging and NeuroDevelopment (MIND; https://www.erasmusmc.nl/en/research/groups/methylation-imaging-and-neurodevelopment-mind-consortium) consortium. The MIND consortium combines research on epigenetics and neuroimaging to better understand the potential role of DNAm in brain development and mental health, and aims to shed light on the relationship between DNAm patterns and brain structure and function across development. It targets each of the key challenges in this new research field by (i) incorporating a developmental perspective; (ii) elucidating the directionality of associations between methylation and the brain via the use of prospective, longitudinal studies across development; and (iii) promoting collaborative science via multi-cohort analyses. We encourage the formation of such collaborative efforts to further understand associations between DNAm and brain structure and function across the life course.

## Conclusion

Neuroimaging epigenetics is a promising, growing field of research building on a recent drive towards more dimensional, multi-system approaches to mental health. To contribute substantially to advancements in mental health research, we advocate for: (1) a greater focus on development; (2) the use of large, prospective, pediatric cohorts with repeated measures of methylation and imaging to assess directionality; and (3) collaborative science to increase comparability across studies.

## Supplementary Material

Supplementary Material

Table S1

Table S2

## Figures and Tables

**Table 1:** Summary of results.

General characteristics	N_all (%)	N_dev (%)	DNAm and brain characteristics	N_all (%)	N_dev (%)	Additional variables examined	N_all (%)	N_dev (%)
**Design**			**DNAm approach**			**Genetic influences**		
Cross-sectional	94 (85%)	13 (57%)	Candidate	74 (67%)	12 (52%)	No	63 (57%)	9 (39%)
Prospective/longitudinal	17 (15%)	10 (43%)	Genome-wide	26 (23%)	7 (30%)	Yes	48 (43%)	14 (61%)
			Epigenetic score	15 (14%)	4 (17%)			
**Sample type**			**DNAm tissue**			**Environmental influences**		
Clinical	50 (45%)	4 (17%)	Peripheral blood	83 (75%)	9 (39%)	No	83 (75%)	10 (43%)
Population/Cohort study	29 (26%)	8 (35%)	Saliva	23 (21%)	11 (48%)	Yes	28 (25%)	13 (57%)
High-risk	20 (18%)	9 (39%)	Cord blood/tissue	5 (5%)	5 (22%)	Of which:		
Genetically-informed (e.g. twin, pedigree)	4 (4%)	2 (9%)	Buccal	5 (5%)	1 (4%)	*Psychological*	23 (82%)	11 (85%)
Other (e.g. convenience, community)	11 (10%)	0 (0%)	Brain (postmortem)	3 (3%)	-	*Physiological*	5 (18%)	2 (15%)
**Developmental period**			**Most investigated candidate genes (>4 studies; *n* = 51)**			**Biological markers**		
Adulthood	85 (77%)	-	*SLC6A4*	17 (15%)	5 (22%)	No	94 (85%)	20 (87%)
Adolescence	16 (14%)	16 (70%)	*OXT/R*	12 (11%)	3 (13%)	Yes	17 (15%)	3 (13%)
Neonatal	6 (5%)	6 (26%)	*FKBP5*	11 (10%)	1 (4%)	Of which:		
Old age	8 (7%)	-	*NR3C1*	6 (5%)	-	*mRNA/gene expression levels*	12 (71%)	2 (67%)
Childhood	6 (5%)	6 (26%)	*BDNF*	6 (5%)	1 (4%)	*Protein levels and metabolomics*	7 (41 %)	1 (33%)
Postmortem	2 (2%)	-						
**Repeated measures**			**Neuroimaging modality**			**Psychiatric and behavioral outcomes**		
No	103 (93%)	20 (87%)	structural MRI	53 (48%)	13 (57%)	No	28 (25%)	10 (43%)
Yes	8 (7%)	3 (13%)	task-based fMRI	39 (35%)	5 (22%)	Yes	83 (75%)	13 (57%)
Of which:			diffusion MRI	21 (19%)	6 (26%)	Of which:		
*Repeated DNAm*	3 (38%)	2 (66%)	resting-state fMRI	8 (7%)	2 (9%)	*Depression and anxiety*	25 (30%)	6 (26%)
*Repeated neuroimaging*	6 (75%)	2 (66%)	PET	6 (5%)	-	*Psychotic disorders*	20 (24%)	2 (9%)
						*Neurodegenerative and ageing-related*	7 (8%)	-
						*Post-traumatic stress disorder*	8 (10%)	-
**Replication**			**Neuroimaging approach**			*Attention deficit hyperactivity disorder*	3 (4%)	1 (4%)
No	100 (99%)	18 (78%)	Region of Interest (ROI)	74 (67%)	16 (70%)	*Cognitive and social processes*	11 (13%)	2 (9%)
Yes	11 (10%)	5 (22%)	Voxel-/ vertex-wise	44 (40%)	8 (35%)	*Substance use and addiction*	7 (8%)	1 (4%)
			Global	14 (13%)	2 (9%)	*Other*	4 (5%)	1 (4%)

Some categories may add up to more than 100% due to certain studies meeting multiple criteria at once (e.g. studies investigating multiple tissues or multiple biological markers). N_all = N for all periods; N_dev = N for the developmental period (<18 years).

**Table 2:** Prospective, pediatric cohorts with repeated measures of methylation and/or imaging.

Cohort	Age at baseline	Years of follow-up	DNAm (number of time points)	MRI (number of time points)	Sample size^a^	Reference
Generation R	Birth	I8y	Y(*n* = 5)	Y (*n* = 4)	Up to *n* = 9778	Kooijman et al. [83]
ALSPAC	Birth	24 y	Y(*n* = 7)	Y(*n* = D	Up to 15,458	Boyd et al. [84]
FinnBrain	Birth	5y	Y(*n* = D	Y (*n* = 2)	Up to 3808	Karlsson et al. [85]
GUSTO	Birth	3y	Y (*n* = 10)	Y (*n* = 3)	Up to 1176	Soh et al. [86]
DCHS	Birth	6y	Y (*n* = 4)	Y (*n* = 3)	Up to 1143	Donald et al. [87]
NEURON	Birth	2y	Y (*n* = 2)	Y (*n* = 2)	Up to 80	Moog et al. [88]
Fragile families	Birth	I5y	Y (*n* = 2)	Y(*n* = D	Up to 2020	Reichmann et al. [89]
Lifespan Baby Connectome Project (planned)	Birth	5y	Y(*n* = D	Y(*n* = 6)	Up to 500	https://www.humanconnectome.org/study/lifespan-baby-connectome-project/overview
IMAGEN	I4y	8y	Y (*n* = 2)	Y (*n* = 3)	Up to 2463	Schumann et al. [90]
Brazilian High-Risk Cohort	6-14 y	6y	Y (*n* = 3)	Y (*n* = 3)	Up to 2512	Salum et al. [91]

a Sample sizes refer to whole cohort, not limited to the subsample with DNAm or MRI.

## Box 1 Glossary of common terms used in neuroimaging epigenetics

### Glossary: Common terms in the field of Neuroimaging Epigenetics

**Neuroimaging epigenetics**: a field of research that aims to uncover the relationship between epigenetic patterns and the brain, and to establish the extent to which epigenetic marks in diverse tissues may enhance our ability to predict, trace or explain genetic and environmental influences on brain and behavior.

### Epigenetics

**DNA methylation (DNAm)**: one of the most commonly studied epigenetic mechanisms, affecting transcriptional activity via the addition of a methyl group to the cytosine base in the DNA (often in the context of a cytosine-guanine (CpG) sequence). It is modulated by both genetic and environmental factors, can be reversible and change dynamically with age [92, 93]. Importantly, DNAm is a driving mechanism in development with key functions for example in embryogenesis [4, 5], cell specification [6], genomic imprinting and X-chromosome inactivation [7, 8], and ageing [9]. Furthermore, disruptions in methylation have been linked to a range of poor health outcomes (including all-cause mortality [94]) and psychiatric disorders [95]. DNAm is highly tissue-specific, which poses a key challenge in the field of neuroimaging epigenetics [47, 48, 50].

**Epigenetic age**: a measure of biological ageing, derived from DNAm. Several of such ageing biomarkers, also referred to as ‘epigenetic clocks’, have been developed. For example, the ‘Horvath clock’ is based on 353 CpGs and can be applied to most tissues types [93], while the ‘Hannum clock’ is specific to blood tissue [96]. Epigenetic age acceleration is most commonly defined as the difference between an individual’s chronological and biological age (i.e. a difference > 0 suggesting an acceleration of biological ageing compared to one’s chronological age).

### Brain imaging

**Magnetic resonance imaging (MRI)**: a method that uses a strong magnetic field and radio waves to produce an image of the brain.

**Structural MRI**: a measure of brain morphology such as cortical thickness, surface area or volume of either the whole brain or individual regions of interest (ROIs).

**Structural connectivity**: based on diffusion MRI or derived measures such as fractional anisotropy, structural connectivity measures water diffusion in brain tissue, giving insights into the structural properties of white matter tracts and aspects of tissue microstructure.

**Functional MRI**: a measure of blood oxygen-level dependent signal in the brain to provide estimates of brain activity during a task or during rest *(resting-state functional connectivity*).

**Position emission tomography (PET)**: a measure of regional metabolic activity, perfusion, or molecular composition in the brain, using radioactive tracers.

## References

[1] Colclough GL, Smith SM, Nichols TE, Winkler AM, Sotiropoulos SN, Glasser MF (2017). The heritability of multi-modal connectivity in human brain activity. Elife.

[2] Jansen AG, Mous SE, White T, Posthuma D, Polderman TJC (2015). What twin studies tell us about the heritability of brain development, morphology, and function: a review. Neuropsychol Rev.

[3] Solmi M, Radua J, Olivola M, Croce E, Soardo L, Salazar de Pablo G (2022). Age at onset of mental disorders worldwide: large-scale meta-analysis of 192 epidemiological studies. Mol Psychiatry.

[4] Smith ZD, Meissner A (2013). DNA methylation: roles in mammalian development. Nat Rev Genet.

[5] Guo H, Zhu P, Yan L, Li R, Hu B, Lian Y (2014). The DNA methylation landscape of human early embryos. Nature.

[6] Bogdanović O, Lister R (2017). DNA methylation and the preservation of cell identity. Curr Opin Genet Dev.

[7] Sharp AJ, Stathaki E, Migliavacca E, Brahmachary M, Montgomery SB, Dupre Y (2011). DNA methylation profiles of human active and inactive X chromosomes. Genome Res.

[8] Elhamamsy AR (2017). Role of DNA methylation in imprinting disorders: an updated review. J Assist Reprod Genet.

[9] Horvath S, Raj K (2018). DNA methylation-based biomarkers and the epigenetic clock theory of ageing. Nat Rev Genet.

[10] Hannon E, Knox O, Sugden K, Burrage J, Wong CCY, Belsky DW (2018). Characterizing genetic and environmental influences on variable DNA methylation using monozygotic and dizygotic twins. PLoS Genet.

[11] Gapp K, Woldemichael BT, Bohacek J, Mansuy IM (2014). Epigenetic regulation in neurodevelopment and neurodegenerative diseases. Neuroscience.

[12] Szyf M, Tang Y-Y, Hill KG, Musci R (2016). The dynamic epigenome and its implications for behavioral interventions: a role for epigenetics to inform disorder prevention and health promotion. Behav Med Pract Policy Res.

[13] Hillary RF, Marioni RE (2021). MethylDetectR: a software for methylation-based health profiling. Wellcome Open Res.

[14] Chen X, Gole J, Gore A, He Q, Lu M, Min J (2020). Non-invasive early detection of cancer four years before conventional diagnosis using a blood test. Nat Commun.

[15] Priesterbach-Ackley LP, Boldt HB, Petersen JK, Bervoets N, Scheie D, Ulhøi BP (2020). Brain tumour diagnostics using a DNA methylation-based classifier as a diagnostic support tool. Neuropathol Appl Neurobiol.

[16] Roy D, Tiirikainen M (2020). Diagnostic power of DNA methylation classifiers for early detection of cancer. Trends Cancer.

[17] Aref-Eshghi E, Kerkhof J, Pedro VP, Barat-Houari M, Ruiz-Pallares N, Andrau J-C (2020). Evaluation of DNA methylation episignatures for diagnosis and phenotype correlations in 42 Mendelian neurodevelopmental disorders. Am J Hum Genet.

[18] Pickles JC, Fairchild AR, Stone TJ, Brownlee L, Merve A, Yasin SA (2020). DNA methylation-based profiling for paediatric CNS tumour diagnosis and treatment: a population-based study. Lancet Child Adolesc Health.

[19] Karimi S, Zuccato JA, Mamatjan Y, Mansouri S, Suppiah S, Nassiri F (2019). The central nervous system tumor methylation classifier changes neuro-oncology practice for challenging brain tumor diagnoses and directly impacts patient care. Clin Epigenet.

[20] Wheater ENW, Stoye DQ, Cox SR, Wardlaw JM, Drake AJ, Bastin ME (2020). DNA methylation and brain structure and function across the life course: a systematic review. Neurosci Biobehav Rev.

[21] Mulder RH, Neumann A, Cecil CAM, Walton E, Houtepen LC, Simpkin AJ (2021). Epigenome-wide change and variation in DNA methylation in childhood: trajectories from birth to late adolescence. Hum Mol Genet.

[22] Tamnes CK, Walhovd KB, Dale AM, Østby Y, Grydeland H, Richardson G (2013). Brain development and aging: overlapping and unique patterns of change. NeuroImage.

[23] Snir S, Farrell C, Pellegrini M (2019). Human epigenetic ageing is logarithmic with time across the entire lifespan. Epigenetics.

[24] Bethlehem RAI, Seidlitz J, White SR, Vogel JW, Anderson KM, Adamson C (2022). Brain charts for the human lifespan. Nature.

[25] Neumann A, Walton E, Alemany S, Cecil C, González JR, Jima DD (2020). Association between DNA methylation and ADHD symptoms from birth to school age: a prospective meta-analysis. Transl Psychiatry.

[26] Provençal N, Arloth J, Cattaneo A, Anacker C, Cattane N, Wiechmann T (2020). Glucocorticoid exposure during hippocampal neurogenesis primes future stress response by inducing changes in DNA methylation. Proc Natl Acad Sci USA.

[27] Walton E, Marioni R, Elliott HR, Cox SR, Deary IJ, Hughes AD (2021). Characterizing the human methylome across the life course: findings from eight UK-based studies. BioRxiv.

[28] Slieker RC, van Iterson M, Luijk R, Beekman M, Zhernakova DV, Moed MH (2016). Age-related accrual of methylomic variability is linked to fundamental ageing mechanisms. Genome Biol.

[29] Talens RP, Christensen K, Putter H, Willemsen G, Christiansen L, Kremer D (2012). Epigenetic variation during the adult lifespan: cross-sectional and longitudinal data on monozygotic twin pairs. Aging Cell.

[30] Gaunt TR, Shihab HA, Hemani G, Min JL, Woodward G, Lyttleton O (2016). Systematic identification of genetic influences on methylation across the human life course. Genome Biol.

[31] Reynolds CA, Tan Q, Munoz E, Jylhävä J, Hjelmborg J, Christiansen L (2020). A decade of epigenetic change in aging twins: Genetic and environmental contributions to longitudinal DNA methylation. Aging Cell.

[32] Teeuw J, Ori APS, Brouwer RM, de Zwarte SMC, Schnack HG, Hulshoff Pol HE (2021). Accelerated aging in the brain, epigenetic aging in blood, and polygenic risk for schizophrenia. Schizophr Res.

[33] Guillaume B, Wang C, Poh J, Shen MJ, Ong ML, Tan PF (2018). Improving mass-univariate analysis of neuroimaging data by modelling important unknown covariates: Application to Epigenome-Wide Association Studies. Neuroimage.

[34] Ou X, Thakali KM, Shankar K, Andres A, Badger TM (2015). Maternal adiposity negatively influences infant brain white matter development: maternal obesity and infant brain. Obesity.

[35] Walton E, Cecil CAM, Suderman M, Liu J, Turner JA, Calhoun V (2017). Longitudinal epigenetic predictors of amygdala:hippocampus volume ratio. J Child Psychol Psychiatry.

[36] Di Sante J, Ismaylova E, Nemoda Z, Gouin J-P, Yu W-J, Caldwell W (2018). Peripheral DNA methylation of HPA axis-related genes in humans: cross-tissue convergence, two-year stability and behavioural and neural correlates. Psychoneuroendocrinology.

[37] McMillan CT, Russ J, Wood EM, Irwin DJ, Grossman M, McCluskey L (2015). C9orf72 promoter hypermethylation is neuroprotective: Neuroimaging and neuropathologic evidence. Neurology.

[38] Swartz JR, Hariri AR, Williamson DE (2017). An epigenetic mechanism links socioeconomic status to changes in depression-related brain function in high-risk adolescents. Mol Psychiatry.

[39] Casey KF, Levesque ML, Szyf M, Ismaylova E, Verner M-P, Suderman M (2017). Birth weight discordance, DNA methylation, and cortical morphology of adolescent monozygotic twins. Hum Brain Mapp.

[40] Chouliaras L, Pishva E, Haapakoski R, Zsoldos E, Mahmood A, Filippini N (2018). Peripheral DNA methylation, cognitive decline and brain aging: pilot findings from the Whitehall II imaging study. Epigenomics.

[41] Davis EG, Humphreys KL, McEwen LM, Sacchet MD, Camacho MC, MacIsaac JL (2017). Accelerated DNA methylation age in adolescent girls: associations with elevated diurnal cortisol and reduced hippocampal volume. Transl Psychiatry.

[42] Freytag V, Carrillo-Roa T, Milnik A, Sämann PG, Vukojevic V, Coynel D (2017). A peripheral epigenetic signature of immune system genes is linked to neocortical thickness and memory. Nat Commun.

[43] Hodgson K, Carless MA, Kulkarni H, Curran JE, Sprooten E, Knowles EE (2017). Epigenetic age acceleration assessed with human white-matter images. J Neurosci.

[44] Raina A, Zhao X, Grove ML, Bressler J, Gottesman RF, Guan W (2017). Cerebral white matter hyperintensities on MRI and acceleration of epigenetic aging: the atherosclerosis risk in communities study. Clin Epigenetics.

[45] Wolf EJ, Logue MW, Hayes JP, Sadeh N, Schichman SA, Stone A (2016). Accelerated DNA methylation age: associations with PTSD and neural integrity. Psychoneuroendocrinology.

[46] Birney E, Smith GD, Greally JM (2016). Epigenome-wide association studies and the interpretation of disease -omics. PLoS Genet.

[47] Lappalainen T, Greally JM (2017). Associating cellular epigenetic models with human phenotypes. Nat Rev Genet.

[48] Michels KB, Binder AM, Dedeurwaerder S, Epstein CB, Greally JM, Gut I (2013). Recommendations for the design and analysis of epigenome-wide association studies. Nat Methods.

[49] Mill J, Heijmans BT (2013). From promises to practical strategies in epigenetic epidemiology. Nat Rev Genet.

[50] Teschendorff AE, Relton CL (2018). Statistical and integrative system-level analysis of DNA methylation data. Nat Rev Genet.

[51] Nagy C, Turecki G (2012). Sensitive periods in epigenetics: bringing us closer to complex behavioral phenotypes. Epigenomics.

[52] Dunn EC, Soare TW, Zhu Y, Simpkin AJ, Suderman MJ, Klengel T (2019). Sensitive periods for the effect of childhood adversity on DNA methylation: results from a prospective, longitudinal study. Biol Psychiatry.

[53] Cecil CAM, Walton E, Smith RG, Viding E, McCrory EJ, Relton CL (2016). DNA methylation and substance-use risk: a prospective, genome-wide study spanning gestation to adolescence. Transl Psychiatry.

[54] Cecil CAM, Lysenko LJ, Jaffee SR, Pingault J-B, Smith RG, Relton CL (2014). Environmental risk, Oxytocin Receptor Gene (OXTR) methylation and youth callous-unemotional traits: a 13-year longitudinal study. Mol Psychiatry.

[55] Aberg KA, Xie LY, McClay JL, Nerella S, Vunck S, Snider S (2013). Testing two models describing how methylome-wide studies in blood are informative for psychiatric conditions. Epigenomics.

[56] Ewald ER, Wand GS, Seifuddin F, Yang X, Tamashiro KL, Potash JB (2014). Alterations in DNA methylation of Fkbp5 as a determinant of blood-brain correlation of glucocorticoid exposure. Psychoneuroendocrinology.

[57] Meng Q, Zhuang Y, Ying Z, Agrawal R, Yang X, Gomez-Pinilla F (2017). Traumatic brain injury induces genome-wide transcriptomic, methylomic, and network perturbations in brain and blood predicting neurological disorders. EBioMedicine.

[58] Wang J, Hodes GE, Zhang H, Zhang S, Zhao W, Golden SA (2018). Epigenetic modulation of inflammation and synaptic plasticity promotes resilience against stress in mice. Nat Commun.

[59] Davey Smith G, Hemani G (2014). Mendelian randomization: genetic anchors for causal inference in epidemiological studies. Hum Mol Genet.

[60] Dekkers KF, van Iterson M, Slieker RC, Moed MH, Bonder MJ, van Galen M (2016). Blood lipids influence DNA methylation in circulating cells. Genome Biol.

[61] Yang Y, Knol MJ, Wang R, Mishra A, Liu D, Luciano M (2022). Epigenetic and integrative cross-omics analyses of cerebral white matter hyperintensities on MRI. Brain.

[62] BIDS|INCF https://www.incf.org/sbp/brain-imaging-data-structure-bids.

[63] COBIDAS|INCF https://www.incf.org/cobidas.

[64] Marees AT, de Kluiver H, Stringer S, Vorspan F, Curis E, Marie-Claire C (2018). A tutorial on conducting genome-wide association studies: quality control and statistical analysis. Int J Methods Psychiatr Res.

[65] Coleman JRI, Euesden J, Patel H, Folarin AA, Newhouse S, Breen G (2016). Quality control, imputation and analysis of genome-wide genotyping data from the Illumina HumanCoreExome microarray. Brief Funct Genomics.

[66] Arnatkeviciute A, Markello RD, Fulcher BD, Misic B, Fornito A (2023). Toward best practices for imaging transcriptomics of the human brain. Biol Psychiatry.

[67] van Rooij J, Mandaviya PR, Claringbould A, Felix JF, van Dongen J, Jansen R (2019). Evaluation of commonly used analysis strategies for epigenome- and transcriptome-wide association studies through replication of large-scale population studies. Genome Biol.

[68] Hannon E, Lunnon K, Schalkwyk L, Mill J (2015). Interindividual methylomic variation across blood, cortex, and cerebellum: implications for epigenetic studies of neurological and neuropsychiatric phenotypes. Epigenetics.

[69] Walton E, Hass J, Liu J, Roffman JL, Bernardoni F, Roessner V (2016). Correspondence of DNA methylation between blood and brain tissue and its application to schizophrenia research. Schizophr Bull.

[70] McDonald CA, Fahey MC, Jenkin G, Miller SL (2018). Umbilical cord blood cells for treatment of cerebral palsy; timing and treatment options. Pediatr Res.

[71] Roura S, Pujal J-M, Gálvez-Montón C, Bayes-Genis A (2015). The role and potential of umbilical cord blood in an era of new therapies: a review. Stem Cell Res Ther.

[72] Walton E, Pingault J-B, Cecil CAM, Gaunt TR, Relton CL, Mill J (2017). Epigenetic profiling of ADHD symptoms trajectories: a prospective, methylome-wide study. Mol Psychiatry.

[73] Hemani G, Zheng J, Elsworth B, Wade KH, Haberland V, Baird D (2018). The MR-base platform supports systematic causal inference across the human phenome. Elife.

[74] Korologou-Linden R, Xu B, Coulthard E, Walton E, Wearn A, Hemani G (2021). The bidirectional causal effects of brain morphology across the life course and risk of Alzheimer’s disease: a cross-cohort comparison and Mendelian randomization meta-analysis. MedRxiv.

[75] Hatcher C, Relton CL, Gaunt TR, Richardson TG (2019). Leveraging brain cortex-derived molecular data to elucidate epigenetic and transcriptomic drivers of complex traits and disease. Transl Psychiatry.

[76] Thompson PM, Andreassen OA, Arias-Vasquez A, Bearden CE, Boedhoe PS, Brouwer RM (2017). ENIGMA and the individual: Predicting factors that affect the brain in 35 countries worldwide. NeuroImage.

[77] Jia T, Chu C, Liu Y, van Dongen J, Papastergios E, Armstrong NJ (2019). Epigenome-wide meta-analysis ofblood DNA methylation and its association with subcortical volumes: findings from the ENIGMA Epigenetics Working Group. Mol Psychiatry.

[78] Walton E, Relton CL, Caramaschi D (2019). Using openly accessible resources to strengthen causal inference in epigenetic epidemiology of neurodevelopment and mental health. Genes.

[79] Peng P, Zhang Y, Ju Y, Wang K, Li G, Calhoun VD (2022). Group sparse joint non-negative matrix factorization on orthogonal subspace for multi-modal imaging genetics data analysis. IEEE/ACM Trans Comput Biol Bioinf.

[80] Bai Y, Pascal Z, Calhoun V, Wang Y-P (2020). Optimized combination of multiple graphs with application to the integration of brain imaging and (epi)genomics data. IEEE Trans Med Imaging.

[81] Majchrzak-Celińska A, Warych A, Szoszkiewicz M (2021). Novel approaches to epigenetic therapies: from drug combinations to epigenetic editing. Genes.

[82] Cecil CAM, Nigg JT (2022). Epigenetics and ADHD: reflections on current knowledge, research priorities and translational potential. Mol Diagn Ther.

[83] Kooijman MN, Kruithof CJ, van Duijn CM, Duijts L, Franco OH, van Ijzendoorn MH (2016). The generation R study: design and cohort update 2017. Eur J Epidemiol.

[84] Boyd A, Thomas R, Hansell AL, Gulliver J, Hicks LM, Griggs R (2019). Data resource profile: the ALSPAC birth cohort as a platform to study the relationship of environment and health and social factors. Int J Epidemiol.

[85] Karlsson L, Tolvanen M, Scheinin NM, Uusitupa H-M, Korja R, Ekholm E (2018). Cohort profile:the FinnBrain Birth Cohort Study (FinnBrain). Int J Epidemiol.

[86] Soh S-E, Chong Y-S, Kwek K, Saw S-M, Meaney MJ, Gluckman PD (2014). Insights from the Growing Up in Singapore Towards Healthy Outcomes (GUSTO) cohort study. Ann Nutr Metab.

[87] Donald KA, Hoogenhout M, du Plooy CP, Wedderburn CJ, Nhapi RT, Barnett W (2018). Drakenstein Child Health Study (DCHS): investigating determinants of early child development and cognition. BMJ Paediatr Open.

[88] Moog NK, Entringer S, Rasmussen JM, Styner M, Gilmore JH, Kathmann N (2018). Intergenerational effect of maternal exposure to childhood maltreatment on newborn brain anatomy. Biol Psychiatry.

[89] Reichman NE, Teitler JO, Garfinkel I, McLanahan SS (2001). Fragile families: sample and design. Child Youth Serv Rev.

[90] Schumann G, Loth E, Banaschewski T, Barbot A, Barker G, Büchel C (2010). The IMAGEN study: reinforcement-related behaviour in normal brain function and psychopathology. Mol Psychiatry.

[91] Salum GA, Gadelha A, Pan PM, Moriyama TS, Graeff-Martins AS, Tamanaha AC (2015). High risk cohort study for psychiatric disorders in childhood: rationale, design, methods and preliminary results. Int J Methods Psychiatr Res.

[92] Ramchandani S, Bhattacharya SK, Cervoni N, Szyf M (1999). DNA methylation is a reversible biological signal. Proc Natl Acad Sci USA.

[93] Horvath S (2013). DNA methylation age of human tissues and cell types. Genome Biol.

[94] Marioni RE, Shah S, McRae AF, Chen BH, Colicino E, Harris SE (2015). DNA methylation age of blood predicts all-cause mortality in later life. Genome Biol.

[95] Barker ED, Walton E, Cecil CAM (2018). Annual research review: DNA methylation as a mediator in the association between risk exposure and child and adolescent psychopathology. J Child Psychol Psychiatry.

[96] Hannum G, Guinney J, Zhao L, Zhang L, Hughes G, Sadda S (2013). Genome-wide methylation profiles reveal quantitative views of human aging rates. Mol Cell.

