# Supplementary material for: A systematic review of neuroimaging epigenetic research: calling for an increased focus on development
Source: Mol Psychiatry. Author manuscript; Available in PMC 2023 Nov 1. (PMC10615743; doi:10.1038/s41380-023-02067-2)
Supplement: Supplementary Material [file EMS178342-supplement-Supplementary_Material.docx]

**Supplementary methods**

**1 Methods**

In this review, we focus on DNAm in relation to the following neuroimaging modalities: structural magnetic resonance imaging (MRI), task-based functional MRI, resting state functional MRI, diffusion MRI and position emission tomography. This study was not pre-registered.

1.1 Search strategy

PubMed/MEDLINE and Embase databases were systematically searched to identify studies that investigated the relationship between DNAm and brain imaging measures. Abstract, titles and keywords were searched for the terms “methylation”, “epigen*”, “MRI”, “magnetic resonance imaging”, “PET”, and “position emission tomography”. We excluded animal studies and studies of cancer and HIV, using the exclusion terms “mouse”, “rodent”, “cancer”, “glioblastoma”, “HIV”. Studies had to be peer-reviewed journal articles in English and published or in press by October 4, 2021; no quality criteria with respect to the study were applied (e.g. study design, statistical power, data generation, data analysis) to obtain a complete overview of the current state of the field. Three authors (VB, CC and EW) independently screened the studies. Any ambiguities were discussed and resolved between the authors. Full search terms were:

**EMBASE search**:

SEARCH QUERY ('methylation':ti,ab,kw OR 'epigen*':ti,ab,kw) AND ('brain imaging':ti,ab,kw OR 'mri':ti,ab,kw OR 'magnetic resonance imaging':ti,ab,kw OR 'pet':ti,ab,kw OR 'positron emission tomography':ti,ab,kw) NOT ('cancer':ti,ab,kw OR 'tumor':ti,ab,kw OR 'glioblastoma':ti,ab,kw) NOT ('mouse':ti,ab,kw OR 'rodent':ti,ab,kw) AND 'english':la AND [1-1-2000]/sd NOT [04-10-2021]/sd AND [2000-2021]/py AND ('article'/it OR 'article in press'/it)

**PUBMED/MEDLINE search**:

(((((((methylation[MeSH Terms] OR methylation[Title/Abstract] OR epigen*[MeSH Terms] OR epigen*[Title/Abstract])) AND ((brain imaging[MeSH Terms] OR brain imaging[Title/Abstract] OR MRI[MeSH Terms] OR MRI[Title/Abstract] OR magnetic resonance imaging[MeSH Terms] OR magnetic resonance imaging[Title/Abstract] OR PET[MeSH Terms] OR PET[Title/Abstract] OR positron emission tomography[MeSH Terms] OR positron emission tomography[Title/Abstract])) NOT ((cancer[MeSH Terms] OR cancer[Title/Abstract] OR tumor[MeSH Terms] OR tumor[Title/Abstract] OR glioblastoma[MeSH Terms] OR gliobalstoma[Title/Abstract])) NOT ((mouse[MeSH Terms] OR mouse[Title/Abstract] OR rodent[MeSH Terms] OR rodent[Title/Abstract])) AND ("2000/01/01"[Date - Publication] : "2021/10/04"[Date - Publication]) AND english[Language] AND "journal article"[Publication Type]

1.2 Study selection

Searches returned n=585 studies in Embase and n=811 studies in PubMed/MEDLINE. An additional 15 studies were identified through other sources (e.g., reference lists, Google Scholar alert), resulting in a total of n=886 studies to be screened after duplicates were removed. Study abstracts were then screened, after which n=733 studies were excluded. This left a total of n=153 full texts to be assessed. Of these, n=42 texts were excluded (for a PRISMA diagram, see SM Figure 1), resulting in a total of n=111 studies to be discussed in the current review.

Full-text articles excluded, with reasons
(N = 4 animal studies

N = 1 cancer study

N = 1 HIV study

N = 3 case studies

N = 6 cohort descriptions

N = 10 no DNAm

N = 4 no brain MRI

N = 5 reviews

N = 1 unrelated topic

N = 1 Poster

N = 6 did not study brain MRI – DNAm association)

Studies included in qualitative synthesis
(n = 111)

Records identified through database searching
(EMBASE n = 585

PUBMED/MEDLINE n = 811)

Full-text articles assessed for eligibility
(n = 153)

Records excluded
(n = 733)

Records screened
(n = 886)

Records after duplicates removed
(n = 886)

Additional records identified through other sources
(n = 15)

## Identification

## Eligibility

## Included

## Screening

SM Figure 1. PRISMA 2009 Flow Diagram

1.3 Data extraction

We extracted the following data from studies that met our eligibility criteria: sample size, sample type (e.g., clinical, population-based, convenience sample), age group (neonates, children, adolescents, adults, elderly), study design (e.g., cross-sectional, longitudinal with respect to the methylation and/or neuroimaging measures), epigenetic approach (genome-wide [e.g., Illumina array focusing on up to 850,000 CpG sites] versus candidate-driven [e.g., candidate gene/s, epigenetic age acceleration (EAA) based on the Horvath (3) or Hannum method (4), see also Box 1]), neuroimaging approach (region-of-interest (ROI)-based versus voxel-wise or global measures; diffusion MRI, PET, resting state functional connectivity, functional MRI, structural MRI [e.g. measures of volume, thickness or surface]), neuroimaging and DNAm preprocessing pipelines, tissue type, covariates, number of time points DNAm or neuroimaging data was collected, whether replication attempts in an independent cohort were made, whether mediation analyses were included, and whether additional biological markers, behavioural outcomes or exposures were measured.
