# Supplementary material for: A systematic review of neuroimaging epigenetic research: calling for an increased focus on development
Source: Mol Psychiatry. Author manuscript; Available in PMC 2023 Nov 1. (PMC10615743; doi:10.1038/s41380-023-02067-2)
Supplement: Table S2 [file EMS178342-supplement-Table_S2.docx]

SM Table 2. Studies with a focus on developmental periods

| **Author** | **DNAm approach / Gene / Tissue** | **Neuroimaging  modality / measure** | **Analysis space** | **ROI / Global** | **Risk / Outcome** | **Design / Sample size** | **Sample type /  Developmental stage** |
| --- | --- | --- | --- | --- | --- | --- | --- |
| Aghajani et al. 2018 | - Candidate - OXTR - Saliva | - tb-fMRI - amplitude analysis,  functional connectivity | ROI,  voxel-wise | basolateral amygdala,  centromedial amygdala | - N - Y – Conduct disorder | - Cross-sectional - 66 | - Clinical - Adolescents/adults |
| Casey et al. 2017 | - Genome-wide (EWAS) - N/A - Saliva | - sMRI - cortical thickness, surface area and volume | voxel-wise |  | - Y - Birth weight - N | - Cross-sectional - 52 pairs | - Genetically-informed  (population twin study) - Adolescents |
| Chen et al. 2015 | - Genome-wide (EWAS) - N/A - Cord blood | - sMRI - WM volume,  GM volume | ROI,  global | - amygdala, caudate,  cerebellum, globus pallidus, hippocampus, thalamus - WM and GM volume, midbrain | - Y – Maternal anxiety - N | - Prospective - 237 | - Population/Cohort study - Neonatal |
| Chiarella et al. 2020 | - Candidate - SLC6A4, FKBP5 - Saliva | - sMRI, rs-fMRI - voxel-based morphometry, functional connectvity, seed analysis | ROI | - 173 regions (rs-MRI) - 12 ROIs (sMRI) | - N - Y - Depression | - Cross-sectional - 45 | - Clinical - Adolescents |
| Davis et al. 2017 | - Epigenetic score (age) - N/A - Saliva | - sMRI - GM volume | ROI | hippocampus, amygdala | - Y – Familial risk for depression - Y - Depression | - Prospective - 46 | - High-risk - Adolescents |
| de Araújo et al. 2020 | - Candidate - TPPP - Saliva | - sMRI, diffusion MRI - GM volume, FA | ROI | corpus callosum | - Y - Childhood trauma - Y - Depression and anxiety | - Prospective - 63 | - High-risk - Children |
| Dean et al. 2021 | - Genome-wide (EWAS) - N/A - Cord blood | - diffusion MRI - FA, intracellular volume fraction, orientation dispersion index | ROI | multiple ROIs | - Y - Maternal depression and anxiety - N | - Cross-sectional - 52 | - High-risk - Neonatal |
| Fujisawa et al. 2019 | - Candidate - OXTR - Saliva | - sMRI - GM volume | ROI | orbitofrontal cortex and dorsal striatum | - Y - Maltreatment - N | - Cross-sectional - 55 | - High-risk - Children/adolescents |
| Guillaume et al. 2018 | - Genome-wide (EWAS) - N/A - Umbilical cord tissue | - diffusion MRI - FA | voxel-wise |  | - N - N | - Prospective - 114 | - Population/Cohort study - Neonatal |
| Hill et al. 2019 | - Candidate - DRD2 - Blood | - sMRI - GM volume | ROI, voxel- / vertex-wise | fusiform, insula, and temporal regions in the left hemisphere | - Y - Familial risk for alcohol dependence - N | - Prospective - 71 | - High-risk - Adolescents/adults |
| Hoare et al. 2020 | - Epigenetic score (age) - N/A - blood | - sMRI, diffusion MRI - GM and WM volumes, cortical thickness and surface area; FA, mean and radial diffusivity | ROI | - multiple ROIs across the whole brain | - Y - Stress - Y - Cognition | - Cross-sectional - 44 | - Cohort study - Adolescents |
| Ismaylova et al. 2018 | - Candidate - SLC6A4 - saliva | - tb-fMRI - amplitude analysis,  functional connectivity | ROI,  voxel-wise | amygdala, orbitofrontal cortex,  anterior cingulate cortex, insula | - N - Y - Social (response to negative stimuli) | - Prospective - 48 pairs | - Geneticall-informed  (newborn twin Study) - Adolescents |
| Kaufman et al. 2018 | - Candidate - OTX2 and 6 other OTX2-regulated genes - saliva | - rs-fMRI - functional connectivity,  seed analysis | voxel-wise |  | - Y - Childhood trauma - Y - Depression | - Cross-sectional - 47 | - High-risk,  convenience sample - Children |
| Marečková et al. 2020 | - Epigenetic score (age) - N/A - saliva, blood | - sMRI - GM volume, GM volume of the frontal, parietal, occipital and temporal lobes | global | GM volume | - N - Y - Anxiety | - Cross-sectional | - Cohort study - Adults (replication in children/adolescents) |
| Nikolova et al. 2014 | - Candidate - SLC6A4 - saliva; replication in blood | - tb-fMRI - amplitude analysis | ROI | amygdala | - N - N | - Cross-sectional - 80 | - Population sample - Adults  (replication in children/adolescents |
| Ong et al. 2019 | - Genome-wide (EWAS) - N/A - cord blood | - diffusion MRI - FA, radial and axial diffusivity | ROI | amygdala and hippocampus | - Y - Prenatal maternal mental health and socioeconomic status - N | - Cross-sectional - 122 | - Population/Birth cohort study - Neonatal |
| Park et al. 2015 | - Candidate - SLC6A4 - blood | - sMRI - cortical thickness | voxel-wise |  | - N - Y - ADHD | Cross-sectional   - 102 | - Clinical - Children/adolescents |
| Ruggeri et al. 2015 | - Candidate - N/A - blood | - tb-fMRI - amplitude analysis | ROI | right inferior frontal gyrus,  right subthalamic nucleus | - N - Y - Substance use (alcohol) | - Cross-sectional - 18 pairs | - Population/Cohort study - Adolescents/adults |
| Sparrow et al. 2016 | - Genome-wide (PCA) - N/A - blood | - diffusion MRI - FA,  mean diffusivity,  tract shape | voxel-wise |  | - Y - Preterm birth - N | - Cross-sectional - 36 | - High-risk - Neonatal |
| Swartz et al. 2017 | - Candidate - SLC6A4 - blood | - tb-fMRI - amplitude analysis | ROI | amygdala | - N - Y - Depression | - Prospective - 132 | - High-risk - Adolescence |
| Teeuw et al. 2021 | - Epigenetic score (age) - N/A - blood | - sMRI - brain age | global | brain age | - N - Y - Schizophrenia | - Prospective - 715 | - Clinical - Adolescents/adults |
| Walton et al. 2017 | - Genome-wide (EWAS) - N/A - blood (cord, whole) | - sMRI - GM volume | ROI | amygdala, hippocampus | - Y - Early-life stress - Y - Schizophrenia | - Prospective - 109 | - Population/Cohort study - Birth/adolescents |
| Wrigglesworth et al. 2019 | - Candidate - BDNF - buccal | - sMRI - cortical thickness | ROI | prefrontal cortex | - Y -Neighbourhood disadvantage - N | - Prospective - 33 | - High-risk - Adolescents |

*Note:* information (such as sample size) specific to the reported analyses on DNAm and MRI data (e.g., the original study might be based on a larger sample or might include a longitudinal design, but not specifically so with respect to DNAm and MRI). WM=white matter, GM=gray matter, ADHD=attention deficit hyperactivity disorder; PCA=principal component analysis; EWAS=epigenome-wide association analysis, sMRI=structural MRI, tb-fMRI=task-based functional MRI, rs-fMRI =resting state functional MRI, FA = fractional anisotropy.
